# Prevention of ventilator-associated pneumonia with probiotics: an overview of systematic reviews

**DOI:** 10.3389/fmicb.2024.1345278

**Published:** 2024-02-14

**Authors:** Minjuan Han, Ke Wang, Lei Sun, Wang Liu, Wanhu Dong

**Affiliations:** ^1^Affiliated Hospital of Shaanxi University of Chinese Medicine, Xianyang, China; ^2^Yan'an University Xianyang Hospital, Xianyang, China; ^3^Shaanxi University of Chinese Medicine, Xianyang, China

**Keywords:** treatment, probiotics, ventilator-associated pneumonia, evidence, adjunctive

## Abstract

**Background:**

Probiotics has been used as an adjuvant therapy for the prevention of ventilator-associated pneumonia (VAP). This study aimed to systematically compile, evaluate, and synthesize previous systematic reviews (SRs) and meta-analyses (MAs) on the prevention of VAP with probiotics.

**Methods:**

The methodological quality, reporting quality, and evidence quality of enrolled studies were, respectively evaluated by Assessment of Multiple Systematic Reviews 2 (AMSTAR-2) tool, Preferred Reporting Items for Systematic Reviews and Meta-Analyses (PRISMA) checklists, and Grading of Recommendations, Assessment, Development, and Evaluation (GRADE) system.

**Results:**

Thirteen eligible publications were analyzed in this overview. The included studies were rated as generally low methodological quality owing to the lack of a registered protocol or a list of exclusion criteria. The inadequate quality of the reports was demonstrated by the lack of reporting on the registration protocols, the lack of reporting on the search strategy, and the lack of reporting on the additional analyses. For GRADE, there were 36.17% (17/47) outcomes graded to be of moderate quality, 42.55% (20/47) to be of low quality, and 21.28% (10/47) to be of very low quality.

**Conclusion:**

Probiotics may be associated with reduced incidence of VAP. However, caution should be exercised when recommending probiotics for the prevention of VAP owing to the poor quality of the current evidence.

## 1 Introduction

Ventilator-associated pneumonia (VAP) is a common pneumonia occurring more than 48 h after endotracheal intubation in the intensive care unit (ICU) (Metersky and Kalil, 2018). The duration of mechanical ventilation (MV) is positively correlated with the incidence of VAP (François et al., 2019). VAP has been reported to occur in approximately 40% of patients experiencing MV (Goutier et al., 2014; Ferrer and Torres, 2018). By prolonging the duration of MV and ICU stays and increasing antibiotic demands, VAP usually negatively impacts the prognosis of critically ill patients (Warren et al., 2003). A recent study performed in Japan revealed that patients with VAP typically spent $67,080 during their hospital stays, a substantial premium above individuals without VAP ($32,196) (Nanao et al., 2021).

Early and widely used approaches included selective oral decontamination or antibiotic-selective GI decontamination in an attempt to reduce the incidence of VAP by manipulating the microbiota using pharmacologic strategies (Maselli and Restrepo, 2011). The current guideline, which was updated 6 years ago, are ambiguous in recommending these two strategies because neither approach has been conclusively proven to have definitive efficacy (Torres et al., 2017). The application of probiotics is a recently emerging strategy that may be beneficial in regulating microbiota imbalances in critically ill patients (Shimizu et al., 2021). Probiotics are commercially available live microbial preparations defined as “living microorganisms that confer health benefits to the host when administered in sufficient amount” (Hill et al., 2014). To date, there have been a number of overlapping systematic reviews (SRs)/meta-analyses (MAs) evaluating the efficacy of probiotics in the prevention of VAP (Siempos et al., 2010; Gu et al., 2012; Wang et al., 2013; Bo et al., 2014; Weng et al., 2017; Chen et al., 2018; Batra et al., 2020; Su et al., 2020; Ji et al., 2021; Zhao et al., 2021; Cheema et al., 2022; Song et al., 2022; Sun et al., 2022). However, evidence from these SRs/MAs has not been uniform. In evidence-based medicine, SRs/MAs are regarded as the highest level of evidence (Chen et al., 2022; Yang et al., 2022). High-quality SRs/MAs contribute to the production of trustworthy evidence, whereas low-quality SRs/MAs may inadvertently influence decisions (Huang et al., 2021, 2022). Therefore, this study aimed to systematically compile, evaluate, and synthesize evidence from previous SRs/MAs on the prevention of VAP.

## 2 Methods

### 2.1 Included and excluded criteria

We considered the following criteria for inclusion: (a) type of literature was limited to SRs/MAs; (b) critically ill patients who received MV; (c) probiotics compared to placebo or usual care; and (d) incidence of VAP, ICU mortality, hospital mortality, duration of MV, length of ICU stay, and length of hospital stay were used as outcomes. We considered the following criteria for exclusion: (a) studies not related to the topic; (b) conference reports, protocols; and (c) no valid data available.

### 2.2 Strategy for searching

Searching was performed in Embase, Web of Science, Cochrane Library, and PubMed. The databases were searched from the time they were first created until November 13, 2023. We used a mix of free keywords and Mesh phrases to perform our search. Keywords used for search included “ventilator-associated pneumonia,” “probiotics,” “systematic review,” and “meta-Analyses.” The PubMed search strategy is displayed in Table 1.

**Table 1:** Search strategy for PubMed.

Query	Search term
# 1	Ventilator-associated pneumonia [Mesh]
# 2	Ventilator-associated pneumonia [Title/Abstract] OR ventilator* [Title/Abstract] OR respirator* [Title/Abstract]
# 3	#1 OR #2
# 4	Probiotics [Mesh]
# 5	Probiotic [Title/Abstract] OR beneficial bacteria [Title/Abstract] OR microecological preparation [Title/Abstract] OR lactobacillus [Title/Abstract] OR streptococcus thermophilus [Title/Abstract] OR bifidobacterium [Title/Abstract] OR clostridium butyricum [Title/Abstract] OR saccharomyces [Title/Abstract] OR bacillus [Title/Abstract]
# 6	#4 OR #5
# 7	Meta-analysis as Topic [Mesh]
# 8	Systematic review [Title/Abstract] OR meta-analyses [Title/Abstract] OR meta analyses [Title/Abstract] OR meta-analysis OR metaanalysis [Title/Abstract]
# 9	#7 OR #8
# 8	#3 AND #6 AND #9

### 2.3 Data collection and extraction

Data collection and extraction were performed by two reviewers independently. Prior to reviewing the complete text of possibly eligible reviews to ascertain whether they matched the inclusion criteria, the abstract and title of the literature were first read. Publication year, authors, nation, risk of bias, interventions, methods of quality evaluation, and a summary of the intervention effects were retrieved for the included reviews.

### 2.4 Methodological evaluation

Assessment of Multiple Systematic Reviews 2 (AMSTAR-2) tool was used independently by two reviewers to assess the methodological quality of the included reviews (Shea et al., 2017). In AMSTAR-2, there are 16 items, of which seven are key items. The items can be rated on three levels: “yes,” “partially yes,” and “no” (Shea et al., 2017).

### 2.5 Reporting quality appraisal

Two reviewers independently assessed the reporting quality of each included review based on Preferred Reporting Items for Systematic Reviews and Meta-Analyses (PRISMA) checklists (Moher et al., 2009). There are 27 checklists in PRISMA, each rated either “no” (not reported), “partially yes” (partially reported), or “yes” (fully reported) (Moher et al., 2009).

### 2.6 Evidence quality evaluation

Two reviewers independently graded the evidence quality of the included reviews using Grading of Recommendations, Assessment, Development, and Evaluation (GRADE) system (Atkins et al., 2004). There are several possible causes of evidence downgrading, including indirectness, inconsistency, publication bias, imprecision, and bias risk (Atkins et al., 2004).

## 3 Results

### 3.1 Selection of literature

**Figure 1:**
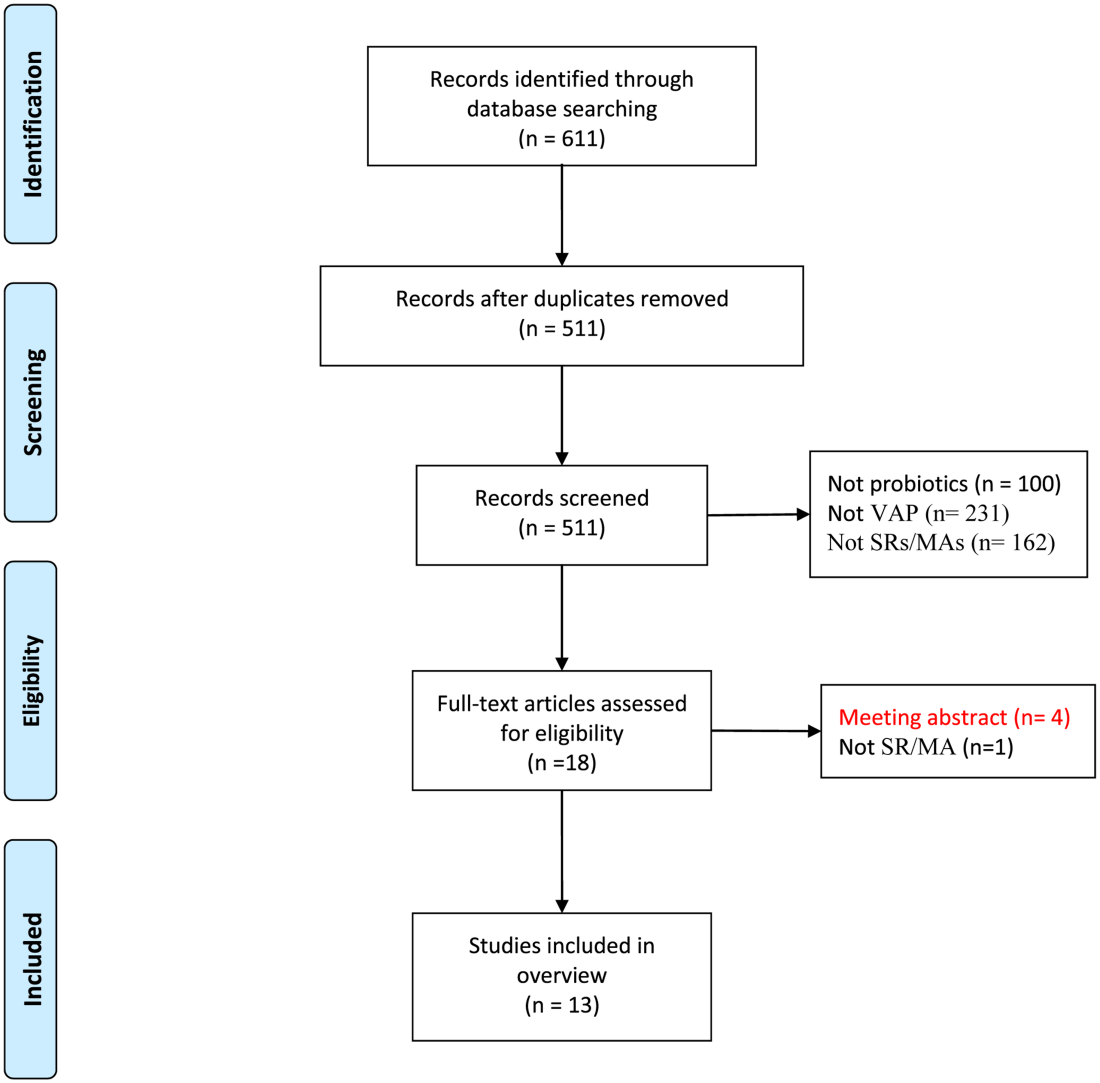
Flow diagram of the literature selection process.

Databases provided 611 potential publications, of which 100 were duplicated. Our review of titles and abstracts revealed 493 records to be excluded. For full-text evaluation, the remaining 18 records were retrieved. As a result, 13 publications (Siempos et al., 2010; Gu et al., 2012; Wang et al., 2013; Bo et al., 2014; Weng et al., 2017; Chen et al., 2018; Batra et al., 2020; Su et al., 2020; Ji et al., 2021; Zhao et al., 2021; Cheema et al., 2022; Song et al., 2022; Sun et al., 2022) were included in this review. The study selection process is depicted in Figure 1.

### 3.2 Study characteristics

Inclusion of SRs/MAs were published between 2010 and 2023 (Table 2). Of the 13 included studies, nine were conducted in China, while the remaining four were conducted in the United States, Brazil, India, and Pakistan. The smallest sample size was found in five trials with 795 cases, while the largest was found in 23 trials with 5,543 cases. Participants in experimental group received probiotics, whereas participants in control group received placebos. Summary of intervention of the included reviews is given in Table 3.

**Table 2:** Characteristics of the included reviews.

Studies	Country	Trials (subjects)	Experimental intervention	Control intervention	Outcomes	Conclusion summary
Siempos et al. (2010)	United States	5 (795)	Probiotics	Placebo	①, ③, ④, ⑤, ⑦	Administration of probiotics is associated with lower incidence of VAP than control.
Gu et al. (2012)	China	7 (1,142)	Probiotics	Placebo	①, ②, ③, ④,⑤, ⑥, ⑦	The limited evidence suggests that probiotics show no beneficial effect in patients who are mechanically ventilated.
Wang et al. (2013)	China	5 (844)	Probiotics	Placebo	①, ②, ③, ⑤, ⑥,	Probiotic prophylaxis of VAP remained inconclusive and it failed to improve the prognosis of general mechanically ventilated patients.
Bo et al. (2014)	Brazil	8 (1,083)	Probiotics	Placebo	①, ②, ③, ④, ⑤,	Evidence suggests that use of probiotics is associated with a reduction in the incidence of VAP.
Weng et al. (2017)	China	13 (1,969)	Probiotics	Placebo	①, ②, ③, ④, ⑤, ⑥	Probiotics could reduce the incidence of VAP in mechanically ventilated patients. It seems likely that probiotics provide clinical benefits for mechanically ventilated patients.
Chen et al. (2018)	China	10 (1,403)	Probiotics	Placebo	①, ②, ③, ④, ⑤	Reduced incidence of VAP in ICU patients given probiotics was found. It seems likely that probiotics provide clinical benefits.
Su et al. (2020)	China	14 (1,975)	Probiotics	Placebo	①, ②, ④, ⑤	The meta-analysis results indicated that the administration of probiotics significantly reduced the incidence of VAP.
Batra et al. (2020)	India	9 (1,172)	Probiotics	Placebo	①,③, ④,⑤, ⑥	Our meta-analysis shows that probiotic administration has a promising role in lowering the incidence of VAP.
Zhao et al. (2021)	China	15 (2,039)	Probiotics	Placebo	①, ⑤	The probiotics helped to prevent VAP without impacting the duration of MV, length of ICU stay or mortality.
Ji et al. (2021)	China	20 (2,428)	Probiotics	Placebo	①, ④, ⑤, ⑥	Probiotics can reduce the incidence of VAP and reduce oropharyngeal and gastric bacterial colonization.
Cheema et al. (2022)	Pakistan	18 (4,893)	Probiotics	Placebo	①, ②, ③, ④, ⑤, ⑥, ⑦	Probiotics may reduce the incidence of VAP but due to the low quality of pooled evidence, the use of probiotics warrants caution.
Sun et al. (2022)	China	23 (5,543)	Probiotics	Placebo	①, ④	The current evidences suggested that prophylactic administration of probiotic might be utilized as a preventive method for VAP in patients who required mechanical ventilation.
Song et al. (2022)	China	15 (4,693)	Probiotics	Placebo	①, ②, ④,⑤	Probiotics are associated with a reduction in VAP, as well as the duration of mechanical ventilation, ICU length of stay, and bacterial colonization.

①: Incidence of VAP; ②: ICU mortality; ③: Hospital mortality; ④: Duration of MV; ⑤: Length of ICU stay; ⑥: Length of hospital stay; ⑦: Adverse events.

**Table 3:** Summary of intervention of the included reviews.

Studies	Probiotic		Comparison		Duration
	Type	Dose	Type	Dose	
Siempos et al. (2010)	*Lactobacillus casei rhamnosus, Lactobacillus plantarum* 299	1 × 10^9^ CFU, 1 × 10^10^ CFU	Placebo	Not applicable	1–2 times daily, 15–28 days
Gu et al. (2012)	*Lactobacillus casei rhamnosu, Lactobacillus plantarum* 299*, Lactobacillus rhamnosus GG*	1–5 × 10^9^ CFU, 1 × 10^10^ CFU	Placebo	Not applicable	1–2 times daily, 15–28 days
Wang et al. (2013)	*Lactobacillus rhamnosus GG, Lactobacillus casei, Lactobacillus acidophilus, Lactobacillus bulgaricus*	2 × 10^9^ CFU, 2 × 10^10^ CFU	Placebo	Not applicable	1–2 times daily, 15–28 days
Bo et al. (2014)	*Lactobacillus casei rhamnosus, Lactobacillus plantarum, Bifidobacterium longum, Lactobacillus bulgaricus, Streptococcus thermophilus*	2 × 10^9^ CFU, 1 × 10^10^ CFU	Placebo	Not applicable	1–3 times daily, 15–28 days
Weng et al. (2017)	*L. casei, Lactobacillus rhamnosus GG, Lactobacillus casei, Lactobacillus acidophilus, Bifidobacterium bifidum*	0.5 × 10^8^ CFU, 1–5 × 10^9^ CFU, 2 × 10^10^ CFU	Placebo	Not applicable	1–2 times daily, 15–28 days
Chen et al. (2018)	*Pediococcus pentoseceus, Leuconostoc mesenteroides, L. paracasei ssp., L. plantaru, Lactobacillus casei rhamnosu, Lactobacillus rhamnosus GG, Lactobacillus acidophilus, Bifidobacterium bifidum*	2 × 10^9^ CFU, 1 × 10^10^ CFU	Placebo	Not applicable	1–2 times daily, 15–28 days
Su et al. (2020)	*L. casei rhamnosus, Lactobacillus plantarum* 299*, Lactobacillus rhamnosus GG, Lactobacillus casei,*	2 × 10^9^ CFU, 1–2 × 10^10^ CFU,1 × 10^11^ CFU	Placebo	Not applicable	1–3 times daily, 14–90 days
Batra et al. (2020)	*Lactobacillus rhamnosus GG, Lactobacillus casei, Lactobacillus acidophilus, Bifidobacterium bifidum*	0.5 × 10^7^ CFU, 0.5 × 10^8^ CFU, 2 × 10^9^ CFU, 2 × 10^10^ CFU	Placebo	Not applicable	1–3 times daily, 14–28 days
Zhao et al. (2021)	*Lactobacillus plantarum* 299*, Lactobacillus rhamnosus GG, Lactobacillus casei, Lactococcus raffinolactis, Lactobacillus acidophilus, Bifidobacterium bifidum*	1–2 × 10^9^ CFU, 1–2 × 10^10^ CFU	Placebo, no placebo	Not applicable	1–3 times daily, 1–4 weeks
Ji et al. (2021)	*Lactobacillus casei, Bifidobacterium, Lactobacillus rhamnosus GG, Enterococcus faecalis*	1–5 × 10^9^ CFU, 1 × 10^10^ CFU	Placebo, no placebo	Not applicable	1–3 times daily, 14–21 days
Cheema et al. (2022)	*Lactobacillus plantarum* 299*, Lactobacillus, Lactobacillus rhamnosus GG, Enterococcus faecalis*	1–3 × 10^9^ CFU, 1 × 10^10^ CFU	Placebo, no placebo	Not applicable	1–3 times daily, 1–4 weeks
Sun et al. (2022)	*Lactobacillus casei, Lacticaseibacillus rhamnosus* Lcr35, *Lactobacillus rhamnosus* GG, *Bifidobacterium breve*, *Lactobacillus plantarum* 299	5 × 10^9^ CFU, 1 × 10^10^ CFU	Placebo	Not applicable	1–2 times daily, 28–60 days
Song et al. (2022)	*Lactobacillus casei, Lactiplantibacillus paraplantarum, Lacticaseibacillus rhamnosus, Bifdobacterium, Streptococcus thermophiles*	2–8 × 10^9^ CFU, 1 × 10^10^ CFU	Placebo, no placebo	Not applicable	1–2 times daily, 1–4 weeks

### 3.3 Methodological evaluation

The AMSTAR-2 assesses one SR/MA as high quality, while the rest were low quality or very low quality (Table 4). Significant deficiencies in methodological quality were demonstrated by the lack of registration protocols (Q2), the lack of exhaustive search strategies (Q4), and the lack of excluded literature lists (Q7). A more detailed explanation can be found in Table 4.

**Table 4:** Quality assessment of the included reviews by the AMSTAR-2 tool.

Author, Year	AMSTAR-2																Quality
	Q1	Q2	Q3	Q4	Q5	Q6	Q7	Q8	Q9	Q10	Q11	Q12	Q13	Q14	Q15	Q16	
Siempos et al. (2010)	Y	N	Y	PY	Y	Y	N	Y	Y	Y	Y	Y	Y	Y	Y	Y	Very low
Gu et al. (2012)	Y	N	Y	Y	Y	Y	N	Y	Y	Y	Y	Y	Y	Y	Y	Y	Very low
Wang et al. (2013)	Y	N	Y	PY	Y	Y	N	Y	Y	Y	Y	Y	Y	Y	Y	Y	Very low
Bo et al. (2014)	Y	Y	Y	Y	Y	Y	Y	Y	Y	Y	Y	Y	Y	Y	Y	Y	High
Weng et al. (2017)	Y	N	Y	PY	Y	Y	N	Y	Y	Y	Y	Y	Y	Y	Y	Y	Very low
Chen et al. (2018)	Y	N	Y	PY	Y	Y	N	Y	Y	Y	Y	Y	Y	Y	Y	Y	Very low
Su et al. (2020)	Y	Y	Y	Y	Y	Y	N	Y	Y	Y	Y	Y	Y	Y	Y	Y	Low
Batra et al. (2020)	Y	Y	Y	Y	Y	Y	N	Y	Y	Y	Y	Y	Y	Y	Y	Y	Low
Zhao et al. (2021)	Y	Y	Y	Y	Y	Y	N	Y	Y	Y	Y	Y	Y	Y	Y	Y	Low
Ji et al. (2021)	Y	Y	Y	Y	Y	Y	N	Y	Y	Y	Y	Y	Y	Y	Y	Y	Low
Cheema et al. (2022)	Y	Y	Y	Y	Y	Y	N	Y	Y	Y	Y	Y	Y	Y	Y	Y	Low
Sun et al. (2022)	Y	N	Y	Y	Y	Y	N	Y	Y	Y	Y	Y	Y	Y	Y	Y	Very low
Song et al. (2022)	Y	Y	Y	Y	Y	Y	N	Y	Y	Y	Y	Y	Y	Y	Y	Y	Low

Y, Yes; PY, Partial yes; N, No.

### 3.4 Reporting quality appraisal

Reporting quality was generally well, with most SRs/MAs following the PRISMA (Table 5). The inadequate quality of the reports was demonstrated by the lack of reporting on the registration protocols (Q5), the lack of reporting on the search strategy (Q8), the lack of reporting on the additional analyses of the methodology section (Q16), and the lack of reporting on the additional analyses of the results section (Q23). A more detailed explanation can be found in Table 5.

**Table 5:** Results of the reporting quality.

Section/topic	Items	Siempos et al. (2010)	Gu et al. (2012)	Wang et al. (2013)	Bo et al. (2014)	Weng et al. (2017)	Chen et al. (2018)	Su et al. (2020)	Batra et al. (2020)	Zhao et al. (2021)	Ji et al. (2021)	Cheema et al. (2022)	Sun et al. (2022)	Song et al. (2022)	Compliance (%)
Title	Q1. Title	Y	Y	Y	Y	Y	Y	Y	Y	Y	Y	Y	Y	Y	100%
Abstract	Q2. Structured summary	Y	Y	Y	Y	Y	Y	Y	Y	Y	Y	Y	Y	Y	100%
Introduction	Q3. Rationale	Y	Y	Y	Y	Y	Y	Y	Y	Y	Y	Y	Y	Y	100%
	Q4. Objectives	Y	Y	Y	Y	Y	Y	Y	Y	Y	Y	Y	Y	Y	100%
Methods	Q5. Protocol and registration	N	N	N	Y	N	N	N	Y	Y	Y	Y	N	Y	46.15%
	Q6. Eligibility criteria	Y	Y	Y	Y	Y	Y	Y	Y	Y	Y	Y	Y	Y	100%
	Q7. Information sources	Y	Y	Y	Y	Y	Y	Y	Y	Y	Y	Y	Y	Y	100%
	Q8. Search	PY	Y	PY	Y	PY	PY	Y	Y	Y	Y	Y	Y	Y	30.77%
	Q9. Study selection	Y	Y	Y	Y	Y	Y	Y	Y	Y	Y	Y	Y	Y	100%
	Q10. Data collection process	Y	Y	Y	Y	Y	Y	Y	Y	Y	Y	Y	Y	Y	100%
	Q11. Data items	Y	Y	Y	Y	Y	Y	Y	Y	Y	Y	Y	Y	Y	100%
	Q12. Risk of bias in individual studies	Y	Y	Y	Y	Y	Y	Y	Y	Y	Y	Y	Y	Y	100%
	Q13. Summary measures	Y	Y	Y	Y	Y	Y	Y	Y	Y	Y	Y	Y	Y	100%
	Q14. Synthesis of results	Y	Y	Y	Y	Y	Y	Y	Y	Y	Y	Y	Y	Y	100%
	Q15. Risk of bias across studies	Y	Y	Y	Y	Y	Y	Y	Y	Y	Y	Y	Y	Y	100%
	Q16. Additional analyses	N	Y	N	Y	Y	N	Y	Y	Y	Y	Y	N	Y	69.23%
Results	Q17. Study selection	Y	Y	Y	Y	Y	Y	Y	Y	Y	Y	Y	Y	Y	100%
	Q18. Study characteristics	Y	Y	Y	Y	Y	Y	Y	Y	Y	Y	Y	Y	Y	100%
	Q19. Risk of bias within studies	Y	Y	Y	Y	Y	Y	Y	Y	Y	Y	Y	Y	Y	100%
	Q20. Results of individual studies	Y	Y	Y	Y	Y	Y	Y	Y	Y	Y	Y	Y	Y	100%
	Q21. Synthesis of results	Y	Y	Y	Y	Y	Y	Y	Y	Y	Y	Y	Y	Y	100%
	Q22. Risk of bias across studies	Y	Y	Y	Y	Y	Y	Y	Y	Y	Y	Y	Y	Y	100%
	Q23. Additional analysis	N	Y	N	Y	Y	N	Y	Y	Y	Y	Y	N	Y	69.23%
Discussion	Q24. Summary of evidence	Y	Y	Y	Y	Y	Y	Y	Y	Y	Y	Y	Y	Y	100%
	Q25. Limitations	Y	Y	Y	Y	Y	Y	Y	Y	Y	Y	Y	Y	Y	100%
	Q26. Conclusions	Y	Y	Y	Y	Y	Y	Y	Y	Y	Y	Y	Y	Y	100%
Funding	Q27. Funding	Y	Y	Y	Y	Y	Y	Y	Y	Y	Y	Y	Y	Y	100%

Y, Yes; PY, Partial yes; N, No.

### 3.5 Evidence quality evaluation

The GRADE system assessed 47 outcomes related to the prevention of VAP with probiotics. There were 36.17% (17/47) outcomes graded to be of moderate quality, 42.55% (20/47) to be of low quality, and 21.28% (10/47) to be of very low quality. Evidence was destroyed primarily because of bias risk, heterogeneity, and imprecision. A more detailed explanation can be found in Table 6.

**Table 6:** Results of evidence quality.

**Review**	**Outcomes**	**№ of trails**	Certainty assessment					№ of patients		Relative effect (95% CI)	Quality
			**Limitations**	**Inconsistency**	**Indirectness**	**Imprecision**	Publication bias	Experimental	Control		
Siempos et al. (2010)	Incidence of VAP	5	Serious^a^	No	No	No	No	316	373	OR 0.61 [0.41, 0.91]	⨁⨁⨁⨁◯
											Moderate
	ICU mortality	4	Serious^a^	No	No	Serious^c^	No	214	267	OR 0.75 [0.47, 1.21]	⨁⨁⨁◯◯
											Low
Gu et al. (2012)	Incidence of VAP	7	Serious^a^	No	No	No	No	576	566	OR 0.82 [0.55, 1.24]	⨁⨁⨁⨁◯
											Moderate
	ICU mortality	4	Serious^a^	No	No	No	No	373	354	OR 0.90 [0.65, 1.27]	⨁⨁⨁⨁◯
											Moderate
	Hospital mortality	4	Serious^a^	No	No	Serious^c^	No	257	256	OR 0.71 [0.48, 1.07]	⨁⨁⨁◯◯
											Low
Wang et al. (2013)	Incidence of VAP	5	Serious^a^	No	No	No	No	423	421	OR 0.94 [0.85, 1.04]	⨁⨁⨁⨁◯
											Moderate
Bo et al. (2014)	Incidence of VAP	8	Serious^a^	No	No	No	No	479	539	OR 0.70 [0.52, 0.95]	⨁⨁⨁⨁◯
											Moderate
	ICU mortality	5	Serious^a^	No	No	Serious^c^	No	325	378	OR 0.84 [0.58, 1.22]	⨁⨁⨁◯◯
											Low
	Hospital mortality	4	Serious^a^	No	No	Serious^c^	No	267	257	OR 0.78 [0.54, 1.14]	⨁⨁⨁◯◯
											Low
	Length of ICU stay	4	Serious^a^	No	No	Serious^c^	No	204	192	MD -1.60 [−6.53, 3.33]	⨁⨁⨁◯◯
											Low
	Duration of MV	2	Serious^a^	No	No	Serious^c^	No	103	100	MD -6.15 [−18.77, 6.47]	⨁⨁⨁◯◯
											Low
Weng et al. (2017)	Incidence of VAP	13	Serious^a^	No	No	No	No	956	1,013	RR 0.73 [0.60, 0.89]	⨁⨁⨁⨁◯
											Moderate
	ICU mortality	6	Serious^a^	No	No	Serious^c^	No	443	495	RR 0.97 [0.74, 1.27]	⨁⨁⨁◯◯
											Low
	Hospital mortality	6	Serious^a^	No	No	Serious^c^	No	440	437	RR 0.81 [0.65, 1.02]	⨁⨁⨁◯◯
											Low
	Length of ICU stay	5	Serious^a^	Serious^b^	No	Serious^c^	No	274	264	MD -2.40 [−6.75, 1.95]	⨁⨁◯◯◯
											Very low
	Length of hospital stay	4	Serious^a^	Serious^b^	No	No ^c^	No	343	339	MD -1.34 [−6.21, 3.54]	⨁⨁⨁◯◯
											Low
Chen et al. (2018)	Incidence of VAP	10	Serious^a^	No	No	No	No	672	731	OR 0.69 [0.54, 0.88]	⨁⨁⨁⨁◯
											Moderate
	ICU mortality	6	Serious^a^	No	No	No	No	443	495	OR 0.95 [0.67, 1.33]	⨁⨁⨁⨁◯
											Moderate
	Hospital mortality	5	Serious^a^	No	No	No	No	385	374	OR 0.86 [0.62, 1.18]	⨁⨁⨁⨁◯
											Moderate
	Length of ICU stay	4	Serious^a^	Serious^b^	No	Serious^c^	No	221	211	MD -1.74 [−6.74, 3.27]	⨁⨁◯◯◯
											Very low
	Duration of MV	2	Serious^a^	Serious^b^	No	Serious^c^	No	109	106	MD -6.21 [−18.83, 6.41]	⨁⨁◯◯◯
											Very low
Su et al. (2020)	Incidence of VAP	13	Serious^a^	No	No	No	No	914	961	OR 0.62 [0.45, 0.85]	⨁⨁⨁⨁◯
											Moderate
	ICU mortality	6	Serious^a^	No	No	No	No	469	524	OR 0.95 [0.67, 1.34]	⨁⨁⨁⨁◯
											Moderate
	Length of ICU stay	10	Serious^a^	Serious^b^	No	No	No	682	736	MD -1.29 [−4.74, 2.15]	⨁⨁⨁◯◯
											Low
	Duration of MV	8	Serious^a^	Serious^b^	No	No	No	569	268	MD -2.37 [−4.67, −0.08]	⨁⨁⨁◯◯
											Low
Batra et al. (2020)	Incidence of VAP	9	Serious^a^	No	No	No	No	564	563	OR 0.70 [0.56, 0.88]	⨁⨁⨁⨁◯
											Moderate
	Length of ICU stay	8	Serious^a^	Serious^b^	No	No	No	538	534	MD -4.20 [−6.73, −1.66]	⨁⨁⨁◯◯
											Low
	Length of hospital stay	4	Serious^a^	Serious^b^	No	Serious^c^	No	324	324	MD -1.94 [−7.17, 3.28]	⨁⨁◯◯◯
											Very low
	Duration of MV	5	Serious^a^	Serious^b^	No	Serious^c^	No	399	400	MD -3.75 [−6.93, −0.58]	⨁⨁◯◯◯
											Very low
Zhao et al. (2021)	Incidence of VAP	9	Serious^a^	No	No	No	No	985	1,054	RR 0.68 [0.60, 0.7]	⨁⨁⨁⨁◯
											Moderate
	Length of ICU stay	8	Serious^a^	Serious^b^	No	Serious^c^	No	706	768	SMD -0.20 [−0.46, 0.06]	⨁⨁◯◯◯
											Very low
	Duration of MV	5	Serious^a^	Serious^b^	No	Serious^c^	No	570	630	SMD -0.20 [−0.41, 0.01]	⨁⨁◯◯◯
											Very low
Ji et al. (2021)	Incidence of VAP	20	Serious^a^	No	No	No	No	1,214	1,214	RR 0.67 [0.59, 0.77]	⨁⨁⨁⨁◯
											Moderate
	Length of ICU stay	15	Serious^a^	Serious^b^	No	Serious^c^	No	984	915	WMD -1.42 [−2.52, −0.31]	⨁⨁◯◯◯
											Very low
	Length of hospital stay	8	Serious^a^	Serious^b^	No	Serious^c^	No	706	768	WMD -1.79 [−3.89, 0.31]	⨁⨁◯◯◯
											Very low
	Duration of MV	12	Serious^a^	Serious^b^	No	Serious^c^	No	783	770	WMD -1.06 [−2.54, 0.43]	⨁⨁◯◯◯
											Very low
Cheema et al. (2022)	Incidence of VAP	18	Serious^a^	Serious^b^	No	No	No	2,489	2,404	RR 0.68 [0.55, 0.84]	⨁⨁⨁◯◯
											Low
	ICU mortality	9	Serious^a^	No	No	No	No	1,908	1,960	RR 0.96 [0.85, 1.09]	⨁⨁⨁⨁◯
											Moderate
	Hospital mortality	8	Serious^a^	No	No	No	No	1,836	1,837	RR 0.94 [0.84, 1.05]	⨁⨁⨁⨁◯
											Moderate
	Length of ICU stay	15	Serious^a^	Serious^b^	No	No	No	984	915	MD -1.42 [−2.52, −0.31]	⨁⨁⨁◯◯
											Low
	Length of hospital stay	9	Serious^a^	Serious^b^	No	No	No	1,957	1,950	MD -1.47 [−4.06, 1.12]	⨁⨁⨁◯◯
											Low
	Duration of MV	12	Serious^a^	Serious^b^	No	No	No	2,093	2,089	MD -1.22 [−3.25, 0.81]	⨁⨁⨁◯◯
											Low
Sun et al. (2022)	Incidence of VAP	23	Serious^a^	Serious^b^	No	No	No	2,824	2,750	RR 0.67 [0.56, 0.81]	⨁⨁⨁◯◯
											Low
Song et al. (2022)	Incidence of VAP	15	Serious^a^	Serious^b^	No	No	No	2,338	2,355	OR 0.58 [0.41, 0.81]	⨁⨁⨁◯◯
											Low
	ICU mortality	7	Serious^a^	No	No	No	No	1,900	1,904	OR 0.94 [0.81, 1.10]	⨁⨁⨁⨁◯
											Moderate
	Length of ICU stay	13	Serious^a^	Serious^b^	No	No	No	2,227	2,244	MD -1.87 [−3.45, −0.28]	⨁⨁⨁◯◯
											Low
	Duration of MV	9	Serious^a^	Serious^b^	No	No	No	1,977	1,995	MD -1.57 [−3.12, −0.03]	⨁⨁⨁◯◯
											Low

IBS-SSS, IBS symptom severity scale; QoL, Quality of life.^a^The experimental design had a large bias in random, distributive findings or was blind; ^b^The confidence interval overlaps less, the heterogeneity test *P* was very small, and the *I*^2^ was larger; ^c^The confidence interval was not narrow enough, or the simple size is too small.

## 4 Discussion

### 4.1 A definitive conclusion cannot be reached

Probiotics as adjuvant therapy for VAP prophylaxis should be used cautiously, as a definitive conclusion cannot be reached on the basis of currently published evidence. First, 11 of the 13 studies suggested that probiotics reduce the incidence of VAP, while the other two (Gu et al., 2012; Wang et al., 2013) studies suggested probiotics failed in the prevention of VAP. It should be noted that the number of patients in each study is quite significant and the number of outcomes for each study is medium high. The contradictory conclusions between these studies seem to be explainable, as the types of probiotics used in these two studies were not consistent with the dose, frequency, and duration of probiotics administered. Therefore, the same probiotic type as well as a standard uniform dosing regimen are more conducive to the comparability of conclusions between different studies. Second, even though 11 of the 13 studies suggested positive results, their unsatisfactory methodological quality limited the credibility of their conclusions. The AMSTAR-2 assesses one SR/MA as high quality, while the rest were low quality or very low quality. In addition, the reporting quality evaluation revealed that the included studies had varying degrees of missing reports in terms of registration protocols, search strategies, and additional analyses. It is well known that convincing evidence from SRs/MAs presupposes that they are sufficiently transparent, scientific and standardized in the production of evidence. Furthermore, the quality of evidence for all outcome indicators was categorized as very low to moderate due to the risk of bias and high degree of heterogeneity, meaning that the effect sizes of these indicators may not exactly match the true picture. Therefore, based on our assessment of the included SRs/MAs, we recommend that probiotics should be used cautiously to prevent VAP.

### 4.2 Research deficiencies to be improved

Deficiencies in methodology, reporting quality, and evidence quality need to be improved during the SR/MA process. Deficiencies in the methodology of SR/MA can reduce the validity of results; weaknesses in the reporting of SR/MA might obscure unfavorable occurrences or overstate the impact of treatments; and inadequately persuasive evidence can cast doubt on the accuracy of findings (McAuley et al., 2000). Evidence can be contaminated by these shortcomings, and clinical decisions can be misled as a result. We found that many of the SRs/MAs we reviewed did not have pre-registered protocols, had inadequate thorough searches, or did not provide an assessment of the design choices made in the studies. It should be noted that only one study (Bo et al., 2014) presents a high-quality assessment by the AMSTAR-2 tool. This study also comments that evidence suggests that use of probiotics is associated with a reduction in the incidence of VAP. Which could confirm the beneficial role of probiotics in VAP. However, this manuscript was published on 2014, which indicates that the missing Q2, Q4, and Q7 in the rest of the articles could have been improved taking this publication as a reference.

### 4.3 Practice and research implications

Registration or publication of study protocols beforehand can reduce any potential bias and enhance process openness. A full search tactic is advantageous to ensuring that the study can be replicated. A list of trials excluded and the reasons for their exclusion is also helpful for reducing publication bias. Sensitivity analyses and subgroup analyses could be taken into account as supplementary analytical techniques when doing data analysis in order to investigate causes of heterogeneity or even to rule out doing a pooled analyses in the event that there is a considerable amount of variation among studies. In particular, high-quality trials published in peer-reviewed journals are a guarantee of high-quality SR/MA. Thus, trials should be designed and conducted according to the Consolidated Standards to ensure high quality evidence and clinical relevance (Shimizu et al., 2021). Furthermore, SR/MA must be designed and implemented in strict compliance with AMSTAR-2 and PRISMA to ensure evidence availability.

### 4.4 Promise of probiotics for VAP

Because of illness and different types of treatment (e.g., broad-spectrum antibiotics for infection management, MV for respiratory failure), it is challenging to maintain a healthy gut microbiota in critically ill patients (Shimizu et al., 2021). In comparison to healthy individuals, patients had 100 times greater levels of *Staphylococcus* and almost 10,000 times fewer total anaerobes, such as *Lactobacillus* and *Bifidobacterium* (Shimizu et al., 2006). When comparing critically ill patients to healthy persons, butyric acid, acetic acid, and organic acids generated from gut microbiota are reduced dramatically (Shimizu et al., 2006). These findings brought to light how the gut microbiota deteriorate during a serious illness (Shimizu et al., 2011). Levels of *Lactobacilli, Bifidobacteria*, and microbial products were significantly higher in patients taking probiotics compared to critically ill patients not taking probiotics (Shimizu et al., 2005). In patients receiving probiotics in ICU, there were significantly greater levels of organic acids, *Lactobacilli,* and *Bifidobacteria* than in those receiving no probiotics (Shimizu et al., 2009). Probiotics are thought to reduce the incidence of VAP as they regulate the composition of the gut microbiota and reduce pathogenic bacterial overgrowth and bacterial translocation through both local and systemic effects, which in turn increase host cell antimicrobial peptides to enhance the immune function (Tegegne and Kebede, 2022). Therefore, the mechanism of probiotics has been interpreted as involving the activity of probiotic metabolites and cellular components to modulate host immunity and inhibit systemic inflammation (Bron et al., 2011).

*Lactobacillus* and *Bifidobacterium* are the main genera of probiotic strains used to prevent VAP, according to the included studies (Siempos et al., 2010; Gu et al., 2012; Wang et al., 2013; Bo et al., 2014; Weng et al., 2017; Chen et al., 2018; Batra et al., 2020; Su et al., 2020; Ji et al., 2021; Zhao et al., 2021; Cheema et al., 2022; Song et al., 2022; Sun et al., 2022). *Lactobacillus plantarum* 299*, Lactobacillus casei, Lactobacillus rhamnosus GG, Enterococcus faecalis, Bifidobacterium bifidum, Lactobacillus bulgaricus, Streptococcus thermophilus, and Lacticaseibacillus rhamnosus* Lcr35 are widely used as probiotics. *Lactobacillus plantarum* 299 was evaluated by six studies, and the results showed that patients who underwent probiotic treatment had a lower incidence of VAP and a significantly shorter length of stay in ICU as well as a shorter duration of MV than the control group. Similarly, *Lactobacillus casei* was evaluated by six studies, and the results showed that patients who underwent probiotic treatment had a lower incidence of VAP, ICU mortality, hospital mortality, and a significantly shorter length of stay in ICU, length of stay in hospital as well as a shorter duration of MV than the control group. Nine studies assessed the effects of *Lactobacillus rhamnosus GG* for VAP, and the pooled analysis revealed considerable benefits in VAP incidence, mortality, length of hospital stay, and duration of MV in patients treated with the probiotic. Two studies evaluated the effects of *Enterococcus faecalis* and *Lactobacillus bulgaricus,* and the pooled results showed that patients receiving probiotics had a lower incidence of VAP and a significantly shorter hospital stay compared to controls. Two studies evaluated outcomes in patients treated with *Bifidobacterium bifidum* and the results indicated that probiotics were also beneficial in patients with VAP. Furthermore, *Streptococcus thermophilus and Lacticaseibacillus rhamnosus* Lcr35 were evaluated by one review, and the pooled analysis revealed considerable benefits in VAP incidence, mortality, length of hospital stay, and duration of MV in patients treated with probiotics. The quality of evidence for both of these widely used probiotics’ effect sizes was graded as low, despite the fact that they both demonstrated therapeutic potential against VAP. As such, care should be taken when suggesting probiotics as preventative therapy for VAP.

## 5 Strengths and limitations

To the best of our knowledge, this study presents the first comprehensive assessment and summary of the data supporting the use of probiotics for the prevention of VAP. However, limitations must be recognized. First, it should be indicated the importance of including the appropriate diet (low in carbohydrates) along with probiotics immediately, to prevent VAP or to avoid the death of the hospitalized patient or large hospital stay. Furthermore, notwithstanding the fact that our evaluation was examined and approved by two separate researchers, different researchers may have differing opinions on any given project due to the subjective nature of quality assessment.

## 6 Conclusion

Probiotics may be associated with reduced incidence of VAP. However, caution should be exercised when recommending probiotics for the prevention of VAP owing to the poor quality of the current evidence.

## Data availability statement

The original contributions presented in the study are included in the article/supplementary material; further inquiries can be directed to the corresponding author.

## Author contributions

MH: Writing – original draft, Writing – review & editing. KW: Conceptualization, Writing – original draft. LS: Conceptualization, Writing – original draft. WL: Conceptualization, Writing – original draft. WD: Visualization, Writing – original draft, Writing – review & editing.
